# Neurorehabilitation as Network Perturbation: Shaping Neuroplasticity with Robotics, Virtual Reality, and Neuromodulation

**DOI:** 10.3390/biomedicines14020411

**Published:** 2026-02-11

**Authors:** Rocco Salvatore Calabrò, Angelo Quartarone

**Affiliations:** IRCCS Centro Neurolesi “Bonino-Pulejo”, 98123 Messina, Italy; angelo.quartarone@irccsme.it

**Keywords:** network neuroscience, neurorehabilitation, neuroplasticity, robotics, virtual reality, neuromodulation, functional connectivity, closed-loop

## Abstract

Neurological injury induces widespread neuroplastic changes that extend well beyond focal structural damage, altering synaptic function, circuit dynamics, and large-scale network organization. While these processes provide the biological substrate for recovery, they can also drive the stabilization of maladaptive network states that constrain long-term functional improvement. Traditional neurorehabilitation has largely emphasized compensation and task practice, often without explicitly targeting the neural dynamics that underlie persistent disability. In this Opinion, we propose that contemporary rehabilitation technologies, including robotics, virtual reality, and neuromodulation, should be conceptualized as mechanistically grounded interventions that actively perturb neural networks and interact with the pathobiology of post-injury reorganization. Drawing on advances in systems and network neuroscience, we examine key molecular, synaptic, and network-level mechanisms that govern adaptive and maladaptive plasticity, and discuss how these technologies modulate error processing, sensory context, and excitability landscapes to reshape recovery trajectories. We argue that when interventions are appropriately structured, timed, and combined within adaptive and closed-loop frameworks, technology-assisted rehabilitation can move beyond compensation and toward principled modulation of neuroplasticity, aligning therapeutic innovation with the biological rules that govern recovery. This perspective highlights the need for network-informed biomarkers and longitudinal approaches to translate technological advances into durable functional gains.

## 1. Introduction

Neurological disorders are among the leading causes of long-term disability worldwide, and their impact is expected to rise as populations age and survival improves after acute events such as stroke and traumatic brain injury [1]. These conditions also encompass chronic neurodegenerative, inflammatory, and genetic diseases that can remodel brain networks over time. Despite significant progress in acute and chronic management, a substantial proportion of individuals experience persistent motor, cognitive, and functional impairments that limit independence and quality of life, underscoring the central role of neurorehabilitation in contemporary medicine. Over the past two decades, advances in neuroscience have profoundly reshaped our understanding of brain injury and recovery. Rather than producing isolated focal deficits, neurological injuries disrupt distributed neural systems, triggering widespread alterations in synaptic function, circuit dynamics, and large-scale network organization. These changes evolve over time and reflect active processes of neuroplastic reorganization that can support recovery but may also stabilize maladaptive patterns of neural activity. Recovery, therefore, is increasingly understood as a network-level phenomenon, shaped by the interaction between injury, experience, and the intrinsic dynamical properties of neural systems [2].

**Figure 1:**
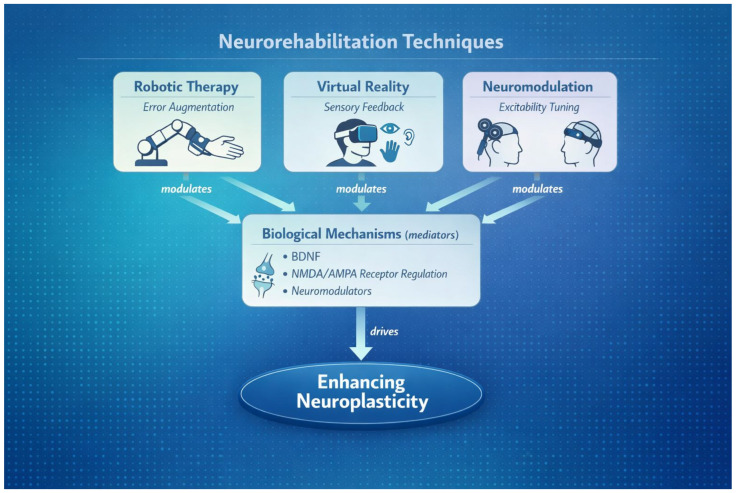
Schematic representation of the principal mechanisms through which technology-assisted rehabilitation shapes post-injury neuroplasticity. Robotic therapy and virtual reality modulate error processing and multisensory integration, respectively, while neuromodulation alters excitability and plastic potential. These interventions converge on shared biological mechanisms, including BDNF signaling, NMDA/AMPA receptor regulation, and neuromodulatory systems (*dopaminergic*, *cholinergic*, *noradrenergic*, and *serotonergic*), collectively biasing synaptic, circuit, and network-level reorganization toward more adaptive recovery trajectories.

In parallel, neurorehabilitation has undergone a technological transformation. Robotics, immersive virtual reality, and neuromodulation now enable unprecedented control over the type, intensity, timing, and context of experience delivered to the injured nervous system. These technologies offer the potential to move beyond dose-based or compensatory approaches by directly engaging the biological mechanisms that govern plasticity, including error processing, multisensory integration, and state-dependent modulation of excitability (Figure 1).

However, technological innovation has often outpaced conceptual integration, with many interventions evaluated primarily in terms of short-term clinical outcomes rather than their capacity to shape neural reorganization in a principled manner.

In this Opinion, we argue that a network neuroscience framework is essential to bridge advances in basic neuroscience and rehabilitation practice. Conceptualizing rehabilitation technologies as structured perturbations of neural systems provides a unifying framework for integrating synaptic plasticity, circuit reorganization, and behavior. This perspective clarifies how different technologies may interact with specific pathomechanisms of plasticity and offers a foundation for the development of mechanism-driven, personalized rehabilitation strategies aimed at guiding recovery trajectories rather than merely compensating for lost function. To facilitate interpretation of this perspective, we first outline how post-injury plasticity can evolve toward either adaptive or maladaptive network states across molecular, cellular, and systems scales. We then discuss how robotics, virtual reality, and neuromodulation can be conceptualized as structured perturbations that manipulate error signals, sensory context, and excitability landscapes to bias reorganization toward more functional configurations. We also address practical limitations and safety considerations that can influence real-world effectiveness. Finally, we propose future directions focused on multimodal integration and closed-loop strategies guided by network-informed biomarkers.

## 2. Neuroplasticity After Brain Injury: Opportunity and Risk

Neurological injury triggers a cascade of plastic changes across molecular, synaptic, and network levels. These changes provide the biological substrate for recovery but do not necessarily converge toward optimal functional configurations. Clinically, this is reflected in the frequent dissociation between early gains and long-term plateaus, even in individuals with preserved tissue and access to rehabilitation. Historically, neurorehabilitation has been framed as a compensatory intervention aimed at maximizing residual abilities rather than modifying the underlying disease process [2].

Advances in systems and network neuroscience challenge this view. Focal lesions disrupt distributed brain networks, inducing changes in functional connectivity, excitability, and inter-regional coordination that evolve over months or years [3,4,5,6]. These alterations are not passive consequences of damage but reflect active reorganization driven by the underlying pathobiology of injury. At this level, reorganization arises from the dynamic interaction of diaschisis, synaptic remodeling, shifts in excitation–inhibition balance, and activity-dependent plasticity within and across spared networks. These processes are further shaped by injury-related neuroinflammatory cascades, glial responses, and changes in neuromodulatory systems, which can either constrain or enable adaptive plasticity depending on timing and behavioral context. Importantly, longitudinal studies demonstrate that post-injury network configurations predict behavioral outcomes more reliably than lesion size alone [7,8], underscoring that recovery is fundamentally a network-level phenomenon rather than a focal one.

This reconceptualization has direct implications for innovation in neurotechnology and rehabilitation. Emerging approaches such as network-guided neuromodulation, closed-loop brain–computer interfaces, and digital phenotyping are uniquely positioned to target and track these evolving network states, rather than static lesion characteristics. By aligning therapeutic interventions with the temporal and mechanistic principles of network reorganization, these technologies offer the potential to shift plasticity toward more adaptive trajectories, moving beyond compensation toward true functional recovery.

Recovery therefore reflects the emergence of new stable network states rather than the restoration of pre-injury organization. Crucially, the nature of these states depends not only on injury characteristics and post-lesional experience but also on individual differences in *motor reserve*—the pre-existing capacity of motor networks to tolerate disruption and reorganize efficiently. This reserve, shaped by lifetime motor experience, structural and functional network redundancy, and genetic and developmental factors, constrains the potential range of plastic responses available after injury. As a result, identical lesions may lead to divergent recovery trajectories, with plasticity manifesting as either adaptive reconfiguration or the stabilization of inefficient, maladaptive neural dynamics. Persistent disability thus arises not solely from irreversible tissue loss but from the consolidation of suboptimal network solutions.

Rehabilitation represents a critical opportunity to interact with these evolving dynamics when interventions are capable of perturbing network organization in a targeted and sustained manner [4,5]. Innovative neurotechnologies—including network-informed neuromodulation, adaptive brain–computer interfaces, and data-driven personalization of therapy—offer the possibility to account for inter-individual variability in motor reserve and to steer plasticity toward more efficient network states. From this perspective, effective recovery is not the normalization of damaged circuits but the guided emergence of functionally viable network configurations aligned with each individual’s latent capacity for reorganization.

## 3. Pathomechanisms of Maladaptive and Adaptive Plasticity

Plasticity after brain injury is governed by mechanisms operating across multiple spatial and temporal scales, spanning molecular, cellular, structural, and large-scale network processes. At the synaptic level, injury profoundly alters neurotransmitter release dynamics, receptor composition, and intracellular signaling pathways, leading to a generalized lowering of thresholds for synaptic modification [9,10]. Changes in glutamatergic and GABAergic transmission, receptor trafficking, and second-messenger cascades promote heightened plastic potential but also increase susceptibility to instability. In this context, homeostatic plasticity mechanisms attempt to stabilize overall levels of neural activity by globally scaling synaptic strength. However, when post-injury experience is asymmetric, impoverished, or poorly structured, these compensatory processes may paradoxically amplify aberrant activity patterns, reinforcing dysfunctional circuit dynamics rather than restoring efficient information processing [11].

**Figure 2:**
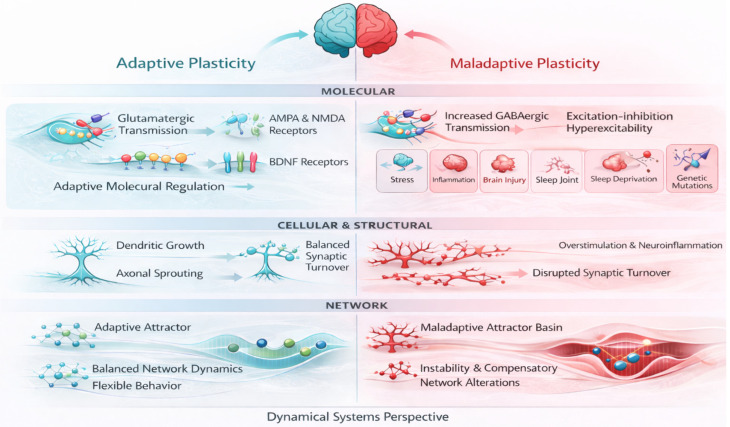
Adaptive and maladaptive neuroplasticity after brain injury across molecular, cellular/structural, and network scales. Adaptive plasticity is associated with balanced AMPA/NMDA signaling, BDNF-dependent remodeling, and flexible network dynamics supporting functional behavior, whereas maladaptive plasticity involves excitation–inhibition imbalance, aberrant structural changes, and stabilization of maladaptive network states leading to compensatory behavior. The molecular scale emphasizes excitation-inhibition balance, including GABAergic tone; the cellular and structural scale depicts dendritic and axonal remodeling; and the network scale reflects changes in integration, segregation, and hub reliance that can stabilize maladaptive states.

Structural plasticity further shapes recovery trajectories by modifying the anatomical substrate on which synaptic changes unfold. Axonal sprouting, dendritic remodeling, and spine turnover occur not only within peri-lesional cortex but also in remote, functionally connected regions, enabling the redistribution of information flow across the injured brain [12]. While such remodeling supports adaptive reorganization under appropriate behavioral constraints, it can equally stabilize maladaptive circuits when behavioral demands favor compensatory strategies over true functional engagement of affected networks (Figure 2).

At the molecular scale, inhibitory signaling may increase as a homeostatic response to excessive excitatory drive, and it can be amplified by changes in interneuron excitability, astrocytic regulation of extracellular GABA, and inflammatory cascades that reshape receptor expression and chloride homeostasis. At the cellular and structural scale, dendritic retraction and spine loss can follow reduced use of affected effectors, excitotoxic stress, and prolonged neuroinflammation, while aberrant sprouting can be promoted by permissive growth signaling in spared pathways in the absence of task constraints that select efficient solutions. At the network scale, these multilevel changes can consolidate patterns such as persistent interhemispheric imbalance, reduced integration within sensorimotor systems, and increased reliance on compensatory hubs, thereby deepening maladaptive attractor basins and reducing the accessibility of more adaptive trajectories.

For example, excessive reliance on the non-paretic limb after stroke may bias experience-dependent plasticity toward the intact hemisphere, strengthening interhemispheric inhibitory imbalance. This, in turn, suppresses activity within the affected motor networks, limits their reintegration into distributed motor circuits, and ultimately constrains long-term recovery potential [13].

At the network level, focal injury induces diaschisis, disrupting long-range communication and altering the organization of critical hubs within distributed brain networks [5,14]. These changes frequently manifest as reduced network integration, excessive segregation of functional modules, or disproportionate reliance on compensatory hubs that support short-term task performance at the expense of flexibility and adaptability. Such configurations may initially sustain behavior but tend to reduce the system’s capacity for efficient reorganization as recovery progresses. Longitudinal neuroimaging studies demonstrate that these altered network states can persist chronically, becoming increasingly stable over time and exerting a strong constraining influence on functional recovery [6,15]. Together, these findings indicate that post-injury plasticity is not inherently beneficial but reflects a competitive process in which neural systems converge toward stable configurations shaped by injury, experience, and intrinsic network constraints.

These observations can be unified within a dynamical systems framework, in which behavior is not localized to discrete brain regions but emerges from trajectories through a high-dimensional neural state space shaped by distributed network interactions [7,16]. Within this framework, neural activity evolves according to the geometry of the state space, which is defined by the structure of connectivity, excitation–inhibition balance, and ongoing neuromodulatory influences. Brain injury perturbs this geometry by altering coupling between network nodes, reshaping attractor landscapes, and biasing the system toward specific patterns of activity. Critically, injury tends to deepen maladaptive attractor basins, increasing their stability and reducing the probability of spontaneous transitions toward more adaptive configurations. As a consequence, even when residual capacity for plasticity is preserved, the system may remain trapped in inefficient dynamical regimes that support limited or compensatory behavior.

From this perspective, recovery is not simply a matter of reactivating damaged circuits but of enabling transitions between network states that are otherwise dynamically inaccessible. Rehabilitation-induced plasticity can therefore be understood as a process that modifies the geometry of the neural state space itself, flattening maladaptive attractors, destabilizing rigid patterns of activity, and expanding the repertoire of accessible trajectories. Such changes require experience that is sufficiently intense, variable, and task-relevant to perturb ongoing dynamics rather than reinforce pre-existing solutions. This framing emphasizes the limitations of interventions that merely strengthen dominant activity patterns and highlights the need for rehabilitation strategies capable of actively driving the system away from entrenched states and toward more flexible, adaptive configurations [4].

## 4. Robotic Rehabilitation as Structured Sensorimotor Perturbation

Robotic rehabilitation systems were initially developed to increase therapy dose, intensity, and reproducibility, particularly in post-stroke motor recovery, where conventional rehabilitation is constrained by therapist availability, fatigue, and variability in delivery [17,18]. While these pragmatic advantages remain central, the mechanistic relevance of robotic rehabilitation extends well beyond repetition or task automation. By enabling precise and programmable manipulation of movement trajectories, interaction forces, temporal structure, and sensory feedback, robotic devices provide a powerful means of shaping the sensorimotor experience that drives plasticity [19]. From a systems perspective, robotics allows controlled exploration of the motor solution space, systematically biasing the neural dynamics that underlie movement generation rather than merely reinforcing existing patterns.

A central mechanism through which robotic systems influence plasticity is error-based learning, a fundamental process underlying motor adaptation and skill acquisition. By amplifying, attenuating, or reshaping movement errors, robotic devices modulate the discrepancy between predicted and actual sensory outcomes, thereby driving synaptic reweighting within corticospinal, cerebellar, and distributed sensorimotor circuits [19,20]. These error signals play a critical role in updating internal models of action, recalibrating feedforward commands, and refining feedback control. When appropriately calibrated, error augmentation increases task salience and destabilizes entrenched motor solutions, facilitating transitions toward more efficient coordination patterns. Conversely, excessive assistance or overly constrained trajectories can reduce afferent drive, blunt error signaling, and reinforce compensatory strategies or learned non-use, ultimately limiting engagement of plasticity mechanisms and constraining recovery potential [20].

Importantly, robotic rehabilitation can be understood within a dynamical systems framework, in which motor behavior reflects trajectories through a high-dimensional neural state space. In this context, robotic devices act as external perturbation tools capable of reshaping the geometry of that space. By manipulating task demands, variability, and error structure, robotics can flatten maladaptive attractor basins, destabilize rigid motor patterns, and expand the repertoire of accessible motor states. However, the efficacy of these perturbations depends critically on their alignment with the individual’s residual capacity for reorganization. Here, the concept of *motor reserve* becomes central: individuals with greater pre-injury network redundancy, flexibility, and learning capacity may respond more robustly to error-driven perturbations, whereas those with limited reserve may require more finely tuned, gradual, or multimodal interventions to avoid consolidation of inefficient dynamics.

Adaptive robotic paradigms represent a critical advance in this regard. By dynamically adjusting assistance, resistance, and task difficulty based on real-time performance, such systems aim to maintain training within an optimal learning zone that is sufficiently challenging to engage plasticity without overwhelming the system. This closed-loop adaptation allows robotic therapy to account for inter-individual variability in motor reserve, fatigue, and learning rate, reducing the risk of under-assistance or over-assistance. Neuroimaging studies indicate that robotic training can induce measurable changes in functional connectivity within motor and sensorimotor networks. These changes have been described as increased coupling between ipsilesional primary motor cortex, premotor regions, and somatosensory cortex, together with a shift toward more integrated and efficient network organization after training [15,21,22]. Recent graph-theoretical analyses further suggest that robot-assisted gait training may promote partial normalization of whole-brain network topology, and these network-level changes can correlate with functional gains [21]. Collectively, these observations support the idea that adaptive robotics can bias reorganization toward more efficient and flexible network configurations when training is task-specific, performance-contingent, and sufficiently demanding.

Taken together, these observations position robotic rehabilitation not merely as a delivery tool for increased practice, but as a platform for precision perturbation of neural dynamics. When embedded within a personalized, network-informed framework, robotic systems have the potential to interact directly with the pathobiology of post-injury plasticity—steering learning, shaping network reorganization, and promoting the emergence of adaptive motor states aligned with each individual’s latent capacity for recovery.

## 5. Virtual Reality and Context-Dependent Plasticity

Virtual reality extends rehabilitation by embedding sensorimotor practice within enriched, multisensory environments that more closely approximate the complexity of real-world interaction [23,24]. Beyond simple task engagement, contextual richness is a powerful modulator of plasticity, as learning is enhanced when sensory feedback, motor intent, and environmental demands are coherently aligned in space and time. Such alignment strengthens associative learning mechanisms and promotes the integration of multisensory information into unified sensorimotor representations. Virtual reality enables systematic and precise manipulation of visual, proprioceptive, and auditory feedback, allowing targeted modulation of the sensory contingencies that shape internal models of the body and action [25]. Through this control, VR provides a means to actively reshape how movements are perceived, predicted, and evaluated by the nervous system.

After brain injury, mismatches between predicted and actual sensory consequences of movement are common and can distort body representations, impair motor planning, and reinforce maladaptive strategies. These prediction errors may stabilize inefficient motor solutions or compensatory behaviors that reduce engagement of affected networks. By recalibrating sensory feedback and restoring coherent sensorimotor contingencies, VR can reduce such mismatches, promoting more accurate sensorimotor predictions and facilitating reorganization across distributed neural systems [25,26]. From a mechanistic standpoint, this recalibration supports the updating of internal models and may help destabilize maladaptive attractor states, enabling transitions toward more adaptive patterns of coordination and control.

Immersive VR environments further engage attentional, motivational, and affective systems that critically modulate learning efficiency. Increased task salience, agency, and embodiment enhance dopaminergic and neuromodulatory influences on plasticity, which are known to gate synaptic change and reinforce learning-related network reconfiguration. These factors are particularly relevant after brain injury, where reduced motivation, attentional deficits, or altered self-representation can limit responsiveness to conventional therapy. By sustaining engagement and promoting a sense of presence and goal-directed interaction, VR may therefore amplify the impact of sensorimotor training on plasticity.

Neuroimaging studies indicate that VR-based interventions influence not only primary sensorimotor regions but also frontoparietal control networks and default-mode network dynamics, supporting broader, systems-level reorganization relevant for functional recovery [27]. Changes in connectivity within and between these networks suggest that VR training can modulate processes related to attention, action monitoring, and self-referential processing, all of which shape behavior and learning. These effects indicate that VR acts not merely on local sensorimotor circuits but on higher-order networks that govern how experience is integrated, evaluated, and consolidated over time. As such, VR-based rehabilitation represents a powerful tool for influencing the neural context in which plasticity unfolds, extending intervention beyond movement execution to the cognitive and perceptual frameworks that support adaptive recovery.

## 6. Neuromodulation as a Plasticity Gatekeeper

Neuromodulation provides a complementary approach to rehabilitation by acting across multiple biological scales, from molecular signaling to large-scale network dynamics, thereby directly shaping the conditions under which plasticity can occur. Non-invasive brain stimulation techniques—most notably transcranial magnetic stimulation (TMS) and transcranial direct current stimulation (tDCS)—differ substantially in their physiological mechanisms, spatial specificity, and interaction with ongoing neural activity [28]. These differences translate into distinct molecular, synaptic, and network-level effects, which in turn determine how stimulation interacts with behavior to influence recovery.

TMS induces focal electric currents through rapidly changing magnetic fields, producing temporally precise neuronal depolarization and synchronized activation of cortical populations. At the molecular and cellular level, TMS engages voltage-gated sodium and calcium channels, leading to action potential generation and calcium influx in stimulated neurons. When applied repetitively, TMS can induce facilitatory or inhibitory after-effects resembling long-term potentiation or depression, depending on stimulation parameters [28]. These effects are mediated by NMDA receptor activation, calcium-dependent intracellular signaling cascades, and downstream modulation of synaptic efficacy and gene expression. In stroke rehabilitation, TMS has been leveraged to counteract pathological interhemispheric inhibition by suppressing contralesional cortical activity or enhancing excitability in perilesional regions of the affected hemisphere [13]. At the network level, these cellular effects translate into transient perturbations of functional connectivity, destabilizing maladaptive synchronization patterns and increasing network flexibility. Such transient destabilization can enhance the responsiveness of the system to concurrent behavioral training, facilitating learning-dependent reorganization [16,29]. However, because these effects are strongly state-dependent and often short-lived when stimulation is delivered in isolation, their translational impact depends critically on precise timing and integration with task-specific rehabilitation [30].

In contrast, tDCS modulates neuronal membrane potentials through weak, sustained electrical currents that shift resting membrane polarization without directly eliciting action potentials. Anodal stimulation generally increases cortical excitability, whereas cathodal stimulation decreases it, although the direction and magnitude of these effects depend strongly on baseline network state and ongoing activity [28]. At the molecular level, tDCS biases synaptic plasticity by modulating NMDA receptor–dependent signaling, intracellular calcium dynamics, and the balance between excitation and inhibition, thereby altering the likelihood that ongoing activity will induce long-term synaptic change [11,26]. Rather than imposing plasticity, tDCS gates it, shaping how experience is translated into synaptic and network reconfiguration. Its relatively broad spatial footprint makes tDCS particularly suitable for modulating distributed networks and global excitability states, but this same property limits its capacity to selectively disrupt deeply entrenched maladaptive attractor configurations when used as a stand-alone intervention.

Peripheral neuromodulation approaches, such as vagus nerve stimulation, further illustrate the translational relevance of neuromodulation by engaging diffuse neuromodulatory systems that regulate plasticity across widespread cortical and subcortical networks [31]. Through activity-dependent release of neuromodulators, these approaches lower plasticity thresholds and enhance signal-to-noise ratios in task-relevant circuits. When stimulation is precisely timed to behavior, plastic changes can be selectively reinforced in active networks, producing durable reorganization. Closed-loop neuromodulation strategies extend this principle by synchronizing stimulation with neural or behavioral events, thereby aligning intervention with moments of heightened plastic susceptibility [32]. From a dynamical systems perspective, neuromodulation acts as a catalyst that transiently lowers energetic barriers between network states, enabling structured rehabilitation to drive transitions away from maladaptive configurations and toward more functional patterns of activity [29,33,34,35].

## 7. Limitations and Future Directions

This perspective reframes recovery as a controllable dynamical process rather than a passive consequence of tissue repair or practice intensity. It also highlights the need to move beyond one-size-fits-all approaches and toward precision rehabilitation strategies that account for individual differences in network architecture, motor reserve, and state-dependent responsiveness. Future advances will depend on the integration of dynamic network biomarkers, adaptive intervention designs, and longitudinal assessment of network evolution. These elements are needed to determine whether technology-assisted rehabilitation can move beyond compensatory strategies and achieve principled modulation of recovery trajectories. Despite their promise, these technologies are not intrinsically beneficial and can introduce predictable risks if deployed without attention to dose, context, and patient-specific constraints. Over-assistive robotic control can reduce active engagement and sensory prediction errors, and it may inadvertently reinforce compensatory kinematics. Immersive virtual reality can elicit fatigue, cybersickness, or headache-like symptoms in a subset of patients, and it requires careful calibration of visual flow and task complexity. Neuromodulation is constrained by contraindications and by substantial inter-individual variability in physiological response, which makes parameter selection and timing critical. Recent syntheses emphasize heterogeneity across protocols and the need for standardized reporting and pragmatic evaluation in real-world settings [36,37].

## 8. Conclusions

Robotics, virtual reality, and neuromodulation are not mutually exclusive, and the most informative next step may be their principled integration within adaptive rehabilitation programs. Virtual environments can provide enriched sensory context and motivation while robotic devices deliver scalable, performance-contingent perturbations. Neuromodulation can be used to bias excitability or neuromodulatory tone at moments of high learning relevance, for example when triggered by behavioral performance or neural state in closed-loop designs [32]. Combining these modalities within a single network-informed framework may increase specificity, reduce the risk of reinforcing maladaptive attractors, and accelerate translation into routine care.

## Data Availability

No new data were created or analyzed in this study. Data sharing is not applicable to this article.
